# Rectal administration of a chlamydial subunit vaccine protects against genital infection and upper reproductive tract pathology in mice

**DOI:** 10.1371/journal.pone.0178537

**Published:** 2017-06-01

**Authors:** Roshan Pais, Yusuf Omosun, Qing He, Uriel Blas-Machado, Carolyn Black, Joseph U. Igietseme, Kohtaro Fujihashi, Francis O. Eko

**Affiliations:** 1 Department of Microbiology, Biochemistry and Immunology, Morehouse School of Medicine, Atlanta, Georgia, United States of America; 2 Veterinary Diagnostic Laboratory, College of Veterinary Medicine, The University of Georgia, Athens, Georgia, United States of America; 3 Centers for Disease Control and Prevention (CDC), Atlanta, Georgia, United States of America; 4 Department of Pediatric Dentistry, Immunobiology Vaccine Center, The University of Alabama at Birmingham, Birmingham Alabama, United States of America; Midwestern University, UNITED STATES

## Abstract

In this study, we tested the hypothesis that rectal immunization with a VCG-based chlamydial vaccine would cross-protect mice against heterologous genital *Chlamydia trachomatis* infection and *Chlamydia*-induced upper genital tract pathologies in mice. Female mice were immunized with a *C*. *trachomatis* serovar D-derived subunit vaccine or control or live serovar D elementary bodies (EBs) and the antigen-specific mucosal and systemic immune responses were characterized. Vaccine efficacy was determined by evaluating the intensity and duration of genital chlamydial shedding following intravaginal challenge with live serovar E chlamydiae. Protection against upper genital tract pathology was determined by assessing infertility and tubal inflammation. Rectal immunization elicited high levels of chlamydial-specific IFN-gamma-producing CD4 T cells and humoral immune responses in mucosal and systemic tissues. The elicited immune effectors cross-reacted with the serovar E chlamydial antigen and reduced the length and intensity of genital chlamydial shedding. Furthermore, immunization with the VCG-vaccine but not the rVCG-gD2 control reduced the incidence of tubal inflammation and protected mice against *Chlamydia*-induced infertility. These results highlight the potential of rectal immunization as a viable mucosal route for inducing protective immunity in the female genital tract.

## Introduction

The majority of *C*. *trachomatis* genital infections worldwide are caused by serovars D, E, and F [1–3] and most infections are asymptomatic. If untreated, *Chlamydia* can ascend to and infect the upper genital tract leading to upper genital tract pathology [4]. Genital *C*. *trachomatis* has been recognized as the most common cause of pelvic inflammatory disease (PID) leading to severe tubal damage, salpingitis, hydrosalpinx and tubal factor infertility (TFI) [5–7]. There is currently no licensed chlamydial vaccine. An effective vaccine should protect against the predominant serovars and prevent development of upper reproductive tract pathology. The severe sequelae associated with chlamydial infection are the consequence of repeated infections caused by poor immunological memory to previous infection. Thus, a vaccine capable of protecting against infection and inducing long lasting immunity is desirable.

We previously showed that intramuscular immunization with a VCG-based chlamydial vaccine expressing the evolutionarily conserved polymorphic outer membrane protein D (PmpD) and porin B (PorB) proteins [8–10] induced long-term, cross protective immune responses in mice [11, 12]. However, the ability of this vaccine to protect against upper genital tract pathology was not evaluated. Mucosal immunization that exploits the tenets of the common mucosal immune system to target immune effectors from one mucosal inductive site to other mucosal effector sites is a practical approach to vaccination against mucosal pathogens like *C*. *trachomatis*. For example, intranasal immunization has been established to provide an effective mucosal route of vaccine delivery against genitally acquired microbial pathogens [13]. However, subunit vaccines often require adjuvants and there are unmitigated concerns about the reactivity of some nasally administered adjuvants that may potentially cause neurological side effects in humans [14]. To overcome this challenge, the rectal route has been proposed as an alternative mucosal route for immunization against diverse microbial pathogens, including Human papilloma virus (HPV) [15], Hepatitis A virus [16], and human immunodeficiency virus (HIV) [17–19]. Moreover, rectal immunization with a Chlamydial ghost-based vaccine was very effective in inducing immunity against enterohaemorrhagic *Escherichia coli* (EHEC) O157:H7 following heterologous challenge [20].

In this study, we tested the hypothesis that rectal (IR) immunization with a subunit chlamydial vaccine would cross-protect mice against heterologous *Chlamydia* genital infection and prevent *Chlamydia*-induced infertility. The results show IR immunization induced cross-reactive immune responses in mucosal and systemic tissues that reduced the length and intensity of genital chlamydial shedding and prevented *Chlamydia*-induced infertility. These results highlight the potential of rectal immunization as a viable route for inducing protective immunity in the female genital tract.

## Materials and methods

### Ethics statement

This study was carried out in strict accordance with the recommendations in the Guide for the Care and Use of Laboratory Animals of the National Institutes of Health. The Institutional Animal Care and Use Committee (IACUC) of Morehouse School of Medicine approved the study protocol (Protocol Number: 16–15). All immunizations, challenge and surgery were performed under ketamine/xylazine anesthesia, and all efforts were made to minimize suffering. Five-week-old female C57BL/6 mice obtained from The Jackson Laboratory (Bar Harbor, ME) were used in this study and were allowed to acclimate for 10 days in the animal facility of Morehouse School of Medicine prior to experimentation.

### Vaccines, *Chlamydia* stocks and antigens

The vaccine candidate used in this study consisted of recombinant VCG expressing the porin B (PorB) and N-terminal portion of polymorphic membrane protein D (PmpD) proteins (rVCG-PmpD/PorB) from *C*. *trachomatis* serovar D. An rVCG construct expressing glycoprotein D from HSV-2, representing a chlamydial irrelevant antigen (rVCG-gD2) was used as antigen control. The rVCG vaccines were produced by protein E-mediated lysis essentially as described previously [21], lyophilized and stored at room temperature until use. In stock preparations of *C*. *trachomatis* serovars D and E used in this study were previously titrated on HeLa cell monolayers followed by purification of elementary bodies (EBs) over renografin gradients and stored at -70^°^C. Chlamydial antigens were prepared by UV-inactivation of EBs for 3 h and stored at -70^°^C until used.

### Experimental design for vaccination and challenge

Groups of mice (10/group) were IR immunized with 50 microliter PBS containing 2 mg of lyophilized rVCG-PmpD/PorB vaccine or rVCG-gD2 control on weeks 0, 2 and 4 as previously described [22] or a single intravaginal inoculation with live *C*. *trachomatis* serovar D EBs (live EB) (1 x 10^6^ IFU/mouse) on week 4. All immunizations were administered while under ketamine (75 mg/kg Ketaset, Zoetis, Florham Park, NJ)/xylazine (15 mg/kg Anased, Lloyd, Shenandoah, IA) anesthesia. One week prior to challenge infection, each mouse was injected with 2.5 mg medroxyprogesterone (Depo Provera; Pharmacia UpJohn Co., Kalamazoo, MI) subcutaneously to synchronize the estrous cycle and increase mouse susceptibility to infection. This treatment is key to a successful mouse model of chlamydial genital infection using human chlamydial strains [23–26]. Mice were then challenged intravaginally with 1.0 x 10^7^ inclusion forming units (IFUs) of *C*. *trachomatis* serovar E to assess cross protection. To evaluate long-term protection, mice were rechallenged intravaginally 10 weeks after the primary challenge infection. Serovar E was chosen for these studies because like serovar D, it is one of the most predominant serovars in human cervical isolates [27]. Mice were observed daily to monitor health status and the level of infection was assessed by enumerating the number of chlamydial inclusion forming units (IFU) from cervicovaginal swabs by indirect immunofluorescence [28] and the mean number of IFU at each time point was calculated.

### Assessment of antigen-specific cellular immune responses

Two weeks after the last immunization, T cells were purified from spleens (SPL) of immunized mice using the gentleMACS Dissociator in combination with the Midi magnetic bead-activated cell sorting (MidiMACS) purification system and CD4-specific MACS microbeads (Miltenyi Biotech, Auburn, CA). *Chlamydia*-specific Th1/Th2 and IL-17 cytokine production by splenic T cells was assessed as described previously [11] using the Bio-Plex cytokine assay kit in combination with the Bio-Plex Manager software (Bio-Rad, Hercules, CA). The concentration of the cytokines in each sample was obtained by extrapolation from a standard calibration curve generated simultaneously. Data were calculated as the mean values (± S.D.) for triplicate cultures for each experiment. The ability of immune CD4 T cells to proliferate in response to *in vitro* restimulation in culture with and without (internal control) chlamydial antigen was assessed using the 5-Bromo-2’-deoxy-uridine (BrdU) T cell proliferation assay and the stimulation index (SI) calculated as described previously [11].

### Assessment of antigen-specific humoral immune responses

The amount of chlamydial PmpD-specific antibodies (IgG2c and IgA) in pooled serum and vaginal wash samples collected 2 weeks postimmunization was measured by a standard ELISA procedure described previously [29]. Briefly, Maxisorb 96-well plates (Costar) were coated overnight with 10 microgram/ml of a synthesized 15-amino acid conserved PmpD peptide (Syd Labs, Malden, MA) in PBS. For generating a standard calibration curve, wells were similarly coated in triplicate with IgA or IgG2c standards (0.0, 12.5, 25, 50. 100, 250, 500 and 1,000 ng/ml). Plates were blocked with 1% bovine serum albumin containing 5% goat serum in PBS and then incubated with 100 microliter of serum or 50 microliter of vaginal wash in twofold serial dilutions. This was followed by incubation with 100 microliter of horseradish peroxidase-conjugated goat anti-mouse IgA or IgG2c (Southern Biotechnology Associates, Birmingham, AL) for 1 h and developed with 2,2’-azino-bis (3-ethylbenzthiazoline-6-sulfonic acid) (ABTS). The optical density was measured at 490 nm on a Spectra Max 250 Microplate Autoreader (Molecular Devices Corp., Sunnyvale, CA). Results, generated simultaneously with the standard curve, display data sets corresponding to absorbance values as mean concentrations (ng/ml) ± standard deviations and represent the mean values from triplicate experiments.

### Fertility assessment

For fertility studies, a separate experiment was performed in which 10 mice/group were similarly immunized with rVCG-PmpD/PorB or rVCG-gD2 control on weeks 0, 2 and 4 and challenged as described above. Ten weeks after the initial primary challenge (12 weeks postimmunization), a time when mice vaginally infected with live chlamydiae are usually susceptible to reinfection, mice were rechallenged with 1.0 x 10^7^ IFU of serovar E per mouse. One week prior to rechallenge, mice received subcutaneous injection of 2.5 mg Depo Provera to synchronize the estrous cycle and increase mouse susceptibility to infection. Four weeks after reinfection, a group of naive uninfected age-matched control and rechallenged mice were mated with proven-fertile males. The mice were visually checked, palpated and weighed daily by the attending veterinary technician to determine pregnancy. Pregnant mice (determined by 3 days of consistent weight gain after caging with males) were sacrificed to evaluate number of embryos. Non-pregnant mice were reintroduced to a different first round proven-breeder male and monitored further and were deemed infertile if this second round mating was unproductive. Subsequently, the total number of pregnant mice and mean number of embryos per group was evaluated.

### Histopathology

Non-pregnant mice from each experimental group above were euthanized and *in situ* gross examination of the genital tract was performed by visual inspection for evidence of upper genital tract pathology. The entire genital tract from the vagina to the ovary was harvested for acquisition of digitized images as described previously [30]. This was fixed in 10% neutral formalin, embedded in paraffin, and serially sectioned longitudinally (5 microns/section) to include the cervix, uterine horns, oviducts and ovaries as well as the lumenal structures. Sections were stained with hematoxylin and eosin (H&E) and scored for severity and distribution of pathologies as well as inflammation, edema, fibrosis, and luminal distension. Pathology severity scores were based on the following scoring system: 0, no significant tissue alterations; 1, minimal; 2, mild; 3, moderate; and 4, severe tissue alterations and distribution scores were based on the following scoring system: 1, focal; 2, multifocal; and 3, diffuse. Sectioning, staining and pathology assessment was performed by a licensed anatomic pathologist (UGA, Athens) who was initially blinded to the identity of the different groups.

### Statistical analysis

Statistical analysis was performed using GraphPad Prism 5.0 software (GraphPad Software, Inc., La Jolla, CA) on a MAC computer. Analysis of variance (ANOVA) was used for all group comparisons. Differences between 2 groups were compared by an unpaired Student t test and Fisher’s exact test was used to compare percentages of fertile mice. Differences between groups were considered statistically significant if *P*-values were ≤ 0.05.

## Results

### IR immunization induces cross protection against challenge with live serovar E chlamydiae

**Fig 1:**
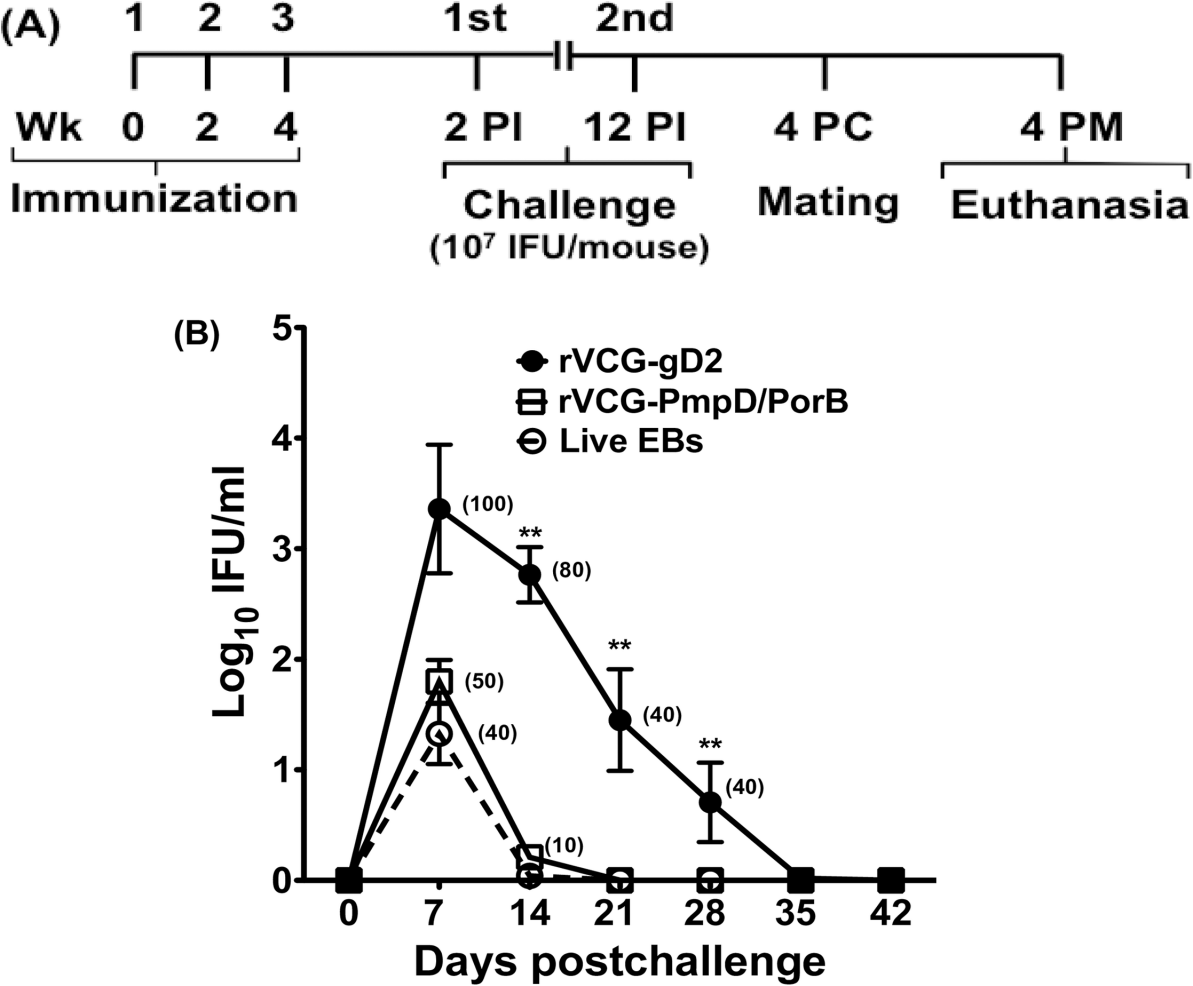
Rectal immunization with rVCG-PmpD/PorB vaccine protects against heterologous challenge with live serovar E chlamydiae. (A) Schematic diagram of the experimental protocol outlining the immunization, challenge and mating schedules. PI, post-immunization; PC, post-challenge; PM, post-mating. (B) Protection against heterologous challenge with live serovar E chlamydiae was evaluated 2 weeks after the last immunization and chlamydial clearance was monitored by enumeration of chlamydiae from cervico-vaginal swabs. The data show the mean recoverable IFUs expressed as log_10_ IFU/ml ± S.D. The experiment was repeated to contain 10 mice per group. Numbers in parentheses are percentages of animals with positive cultures at each time point.

Vaccine efficacy was evaluated by challenging rectally immunized animals intravaginally with live chlamydiae two weeks after the last immunization according to the experimental protocol shown in Fig 1A. Infections were monitored by weekly cervicovaginal swabbing of individual animals and numbers of inclusions were visualized and enumerated in HeLa cell monolayers by indirect immunofluorescence. Fig 1B shows that IR immunization with rVCG-PmpD/PorB or live chlamydial EBs resulted in significant reduction in the number of mice shedding chlamydiae and a lower intensity and duration of chlamydial shedding compared to controls. By day 14 postchallenge, all the live EB-immunized mice and all but one rVCG-PmpD/PorB-immunized mouse had resolved the infection whereas 80% of the controls still shed large numbers of chlamydiae at this time point (Fig 1B).

### Induction of antigen-specific Th1/Th2 cytokine responses induced by rVCG-PmpD/PorB

**Fig 2:**
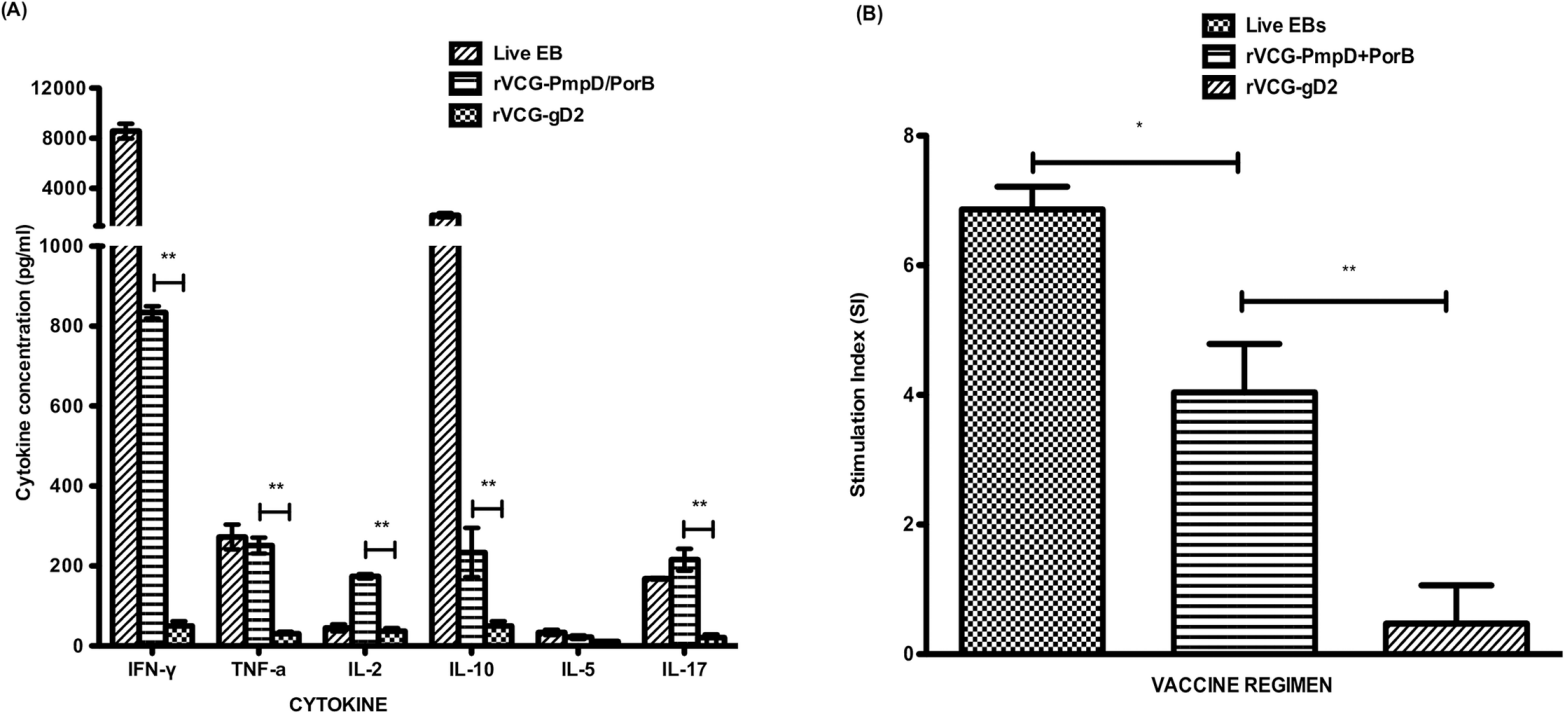
Antigen-specific T cell-mediated immune responses. Pooled CD4+ T cells purified from the spleens of immunized mice and controls were restimulated *in vitro* with *C*. *trachomatis* serovar E antigen (UV-irradiated EBs; 10 microgram/ml). The amount of cross-reactive chlamydial-specific Th1 (IFN-gamma, TNF-alpha, IL-2) and Th2 (IL-5, IL-10) as well as IL-17 cytokines contained in supernatants of culture-stimulated CD4+ T cells was measured using Bio-Plex cytokine assay kit. The concentration of the cytokines in each sample was obtained by extrapolation from a standard calibration curve generated simultaneously. Data were calculated as the mean values (± S.D.) for triplicate cultures for each experiment. The controls (cultures without antigen) did not contain detectable levels of cytokine and so the data were excluded from the results. The results are from two independent experiments and are shown as mean cytokine concentrations (pg/ml) ± SD (A). Significant differences between Th1 and Th2 cytokines (IFN-gamma and IL-5) are indicated by asterisk (*P* <0.05). Antigen-specific CD4+T cell proliferative responses were assessed for their ability to proliferate in response to *in vitro* restimulation in culture with chlamydial serovar E antigen. Results are expressed as stimulation index (SI) values (B), the ratio between absorbance values of stimulated and non-stimulated cells and the bars represent the mean and S.D. of three independent experiments. *p<0.05 (rVCG-PmpD/PorB vaccine versus rVCG-gD2 control and rVCG-PmpD/PorB vaccine versus live EBs).

To assess specific Th1/Th2 cell responses induced by the vaccine candidates, CD4+ T cells were purified from the spleens of immunized mice 2 weeks postimmunization and analyzed for Th1/Th2 cytokine secretion upon restimulation with *C*. *trachomatis* serovar E antigen (UV-irradiated serovar E EBs). Significantly higher (*p*< 0.05) amounts of the Th1 cytokines, IFN-gamma, TNF-α and IL-2, and IL-17 were produced by immune CD4 T cells from live EB- and rVCG-PmpD/PorB-immunized mice compared to those from gD2 control mice (Fig 2A). Although significantly higher (*p*< 0.05) amounts of the Th2 cytokine, IL-10 were secreted by vaccine-induced immune T cells, there was no difference in the levels of IL-5 (Th2 cytokine) produced by T cells from vaccine and control immunized mice. Taken together, the results specify a cross-reactive antigen-specific Th1-type immune response. Immune CD4 T cells were also assessed for their ability to proliferate in response to *in vitro* restimulation in culture with serovar E chlamydial antigen. As expected, immune CD4 T cells from live *Chlamydia*- and rVCG-PmpD/PorB-immunized mice proliferated in response to serovar E EBs. Fig 2B shows the SI value of T cells from live EB-immunized mice was significantly higher (p< 0.05) than that of T cells from rVCG-PmpD/PorB-immunized mice. However, immune CD4 T cells from gD2-immunized mice did not proliferate in response to serovar E EBs. The results suggest IR immunization activated T cells to proliferate in response to restimulation with serovar E chlamydial antigen with the live chlamydial EBs showing an immunogenic advantage.

### Induction of cross-reactive PmpD-specific antibody responses

**Fig 3:**
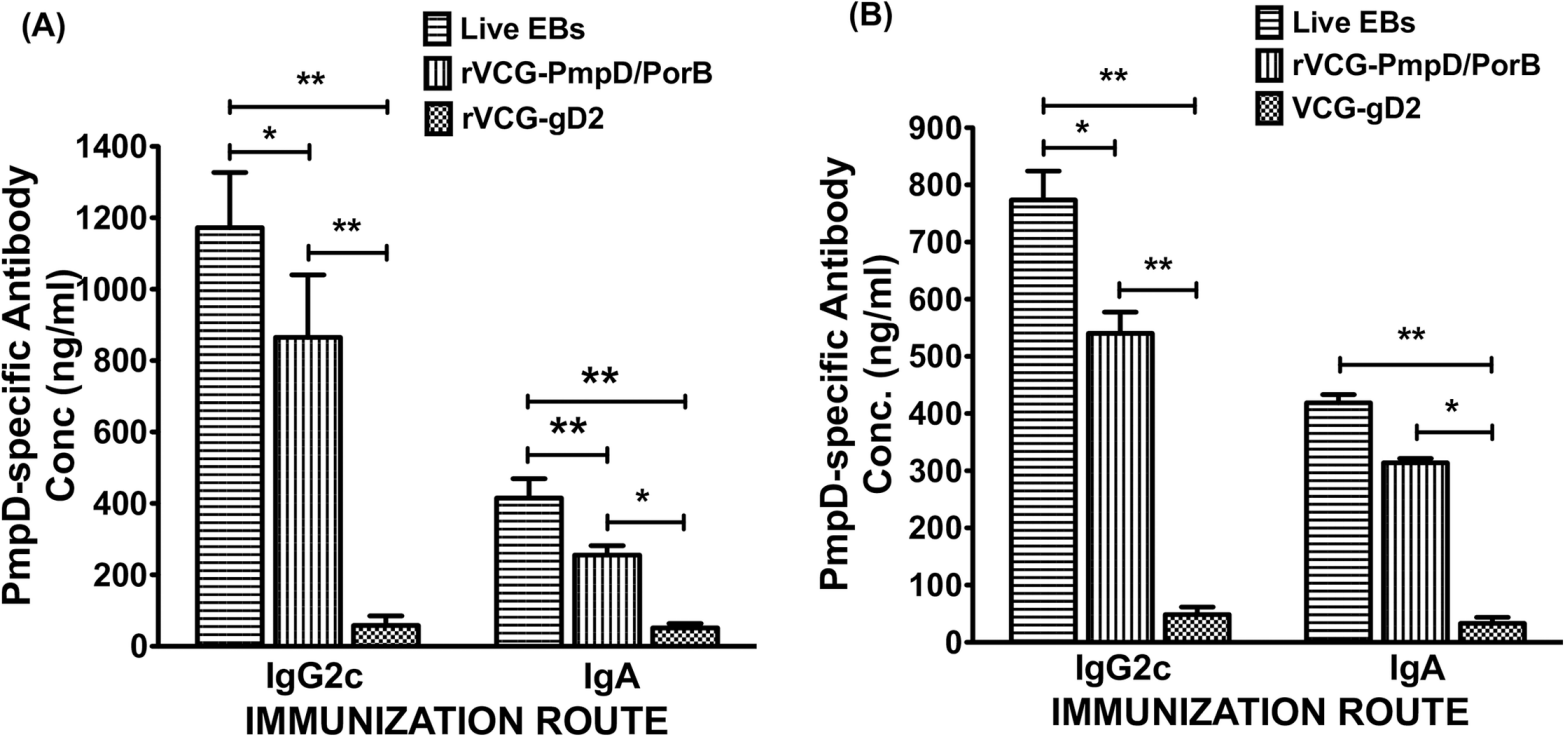
Rectal immunization with rVCG-PmpD/PorB vaccine elicits chlamydial PmpD-specific IgA and IgG2c antibody responses 2 weeks postimmunization. The concentration of elicited antibodies in each immunized group was assessed from pooled serum and vaginal lavage samples obtained 2 weeks postimmunization by antibody ELISA. Results generated simultaneously with a standard curve, display data sets corresponding to absorbance values as mean concentrations (ng/ml) ± SD of triplicate cultures for each experiment. The results are from two independent experiments and show chlamydial PmpD-specific IgA and IgG2c antibody responses from serum (A) and vaginal wash samples (B). *Statistically significant (*p*< 0.05) differences between vaccinated groups.

PmpD-specific antibody responses elicited 2 weeks after immunization were measured by antibody ELISA assay. IR immunization with rVCG-PmpD/PorB and live *Chlamydia* EBs induced significant (*P*< 0.05) PmpD-specific IgG2c and IgA antibodies in both serum (Fig 3A) and vaginal secretions (Fig 3B) compared to controls, with *Chlamydia* EB-elicited levels being significantly higher (p< 0.05). In both serum and genital secretions, IgG2c levels were significantly higher (*p*< 0.05) compared to IgA levels. The results indicate that IR immunization induces anti-chlamydial antibodies in both systemic and mucosal tissues, with IgG2c being consistently higher compared to IgA in both compartments.

### IR immunization confers long lasting cross protection against reinfection with live serovar E chlamydiae

**Fig 4:**
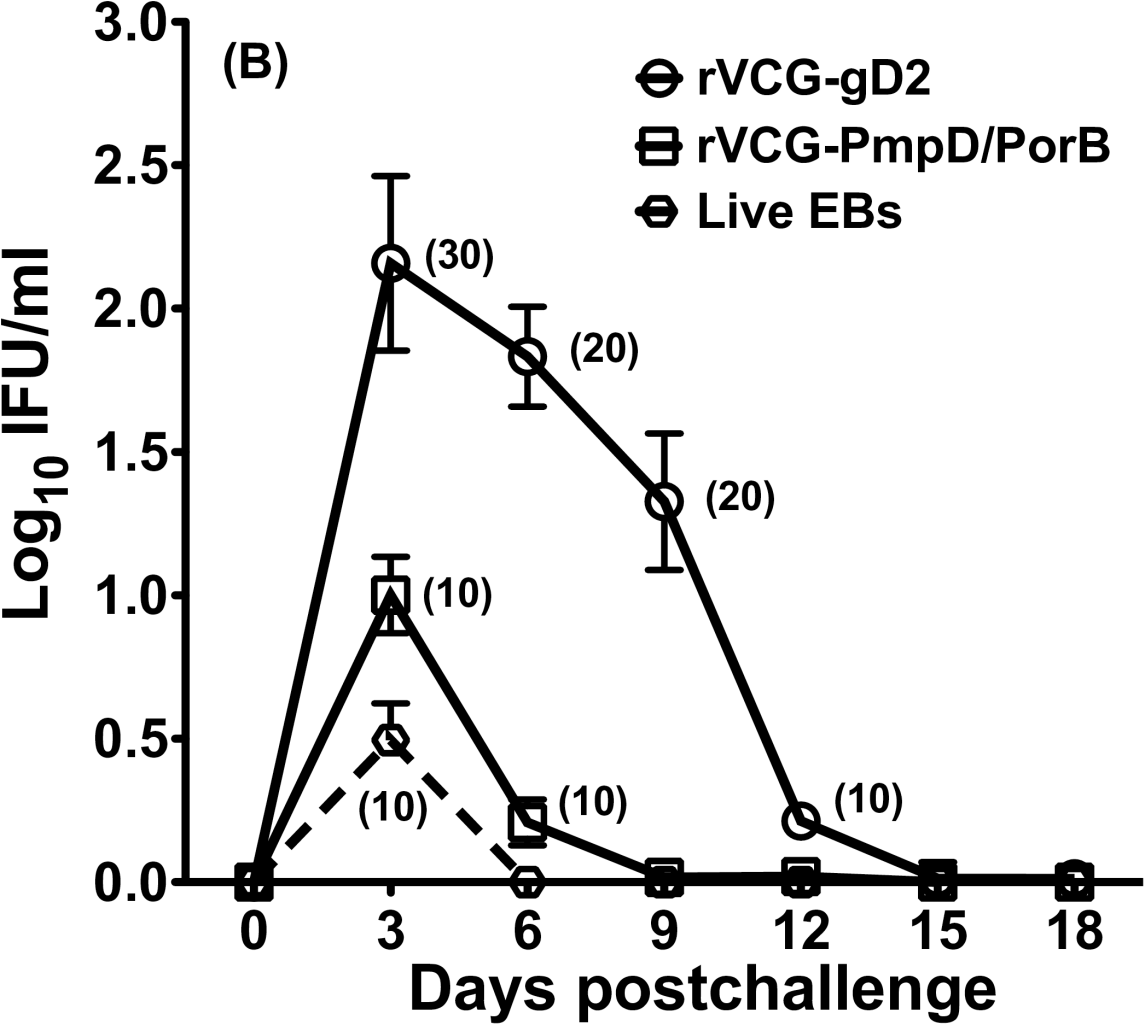
Rectal immunization with rVCG-PmpD/PorB vaccine protects against reinfection with live serovar E chlamydiae. Protection against heterologous reinfection with live serovar E chlamydiae was evaluated 10 weeks after primary challenge. Chlamydial burden was monitored by enumeration of chlamydiae from cervico-vaginal swabs every three days and the number of IFUs/ml recovered from genital swabs of mice was calculated. Each data point represents the mean ± SD of the individual number of recoverable IFUs from each mouse/group of 10 animals collected at the indicated time points expressed as log_10_ IFU/ml ± S.D. Numbers in parentheses are percentages of animals with positive cultures at each time point.

Following resolution of the primary genital tract infection, vaccine- and control-immunized mice were rechallenged intravaginally 10 weeks after the primary challenge infection. Chlamydial clearance was monitored by enumeration of chlamydiae from cervico-vaginal swabs. The result showed a significant (*p*< 0.05) reduction in the number of mice shedding chlamydiae as well as in the intensity (numbers of IFUs shed) and duration of chlamydial shedding in both immunized and control groups following rechallenge (Fig 4). By day 9 after rechallenge, all rVCG-PmpD/PorB- and live EB (serovar D)-immunized mice had completely cleared the infection. By day 15 post rechallenge, even the gD2-immunized mice had completely cleared the challenge infection. These results indicate that IR immunization with rVCG-PmpD/PorB induces immune memory that results in faster clearance of infection following rechallenge.

### IR immunization with rVCG-PmpD/PorB protects mice against *Chlamydia*-induced infertility

**Fig 5:**
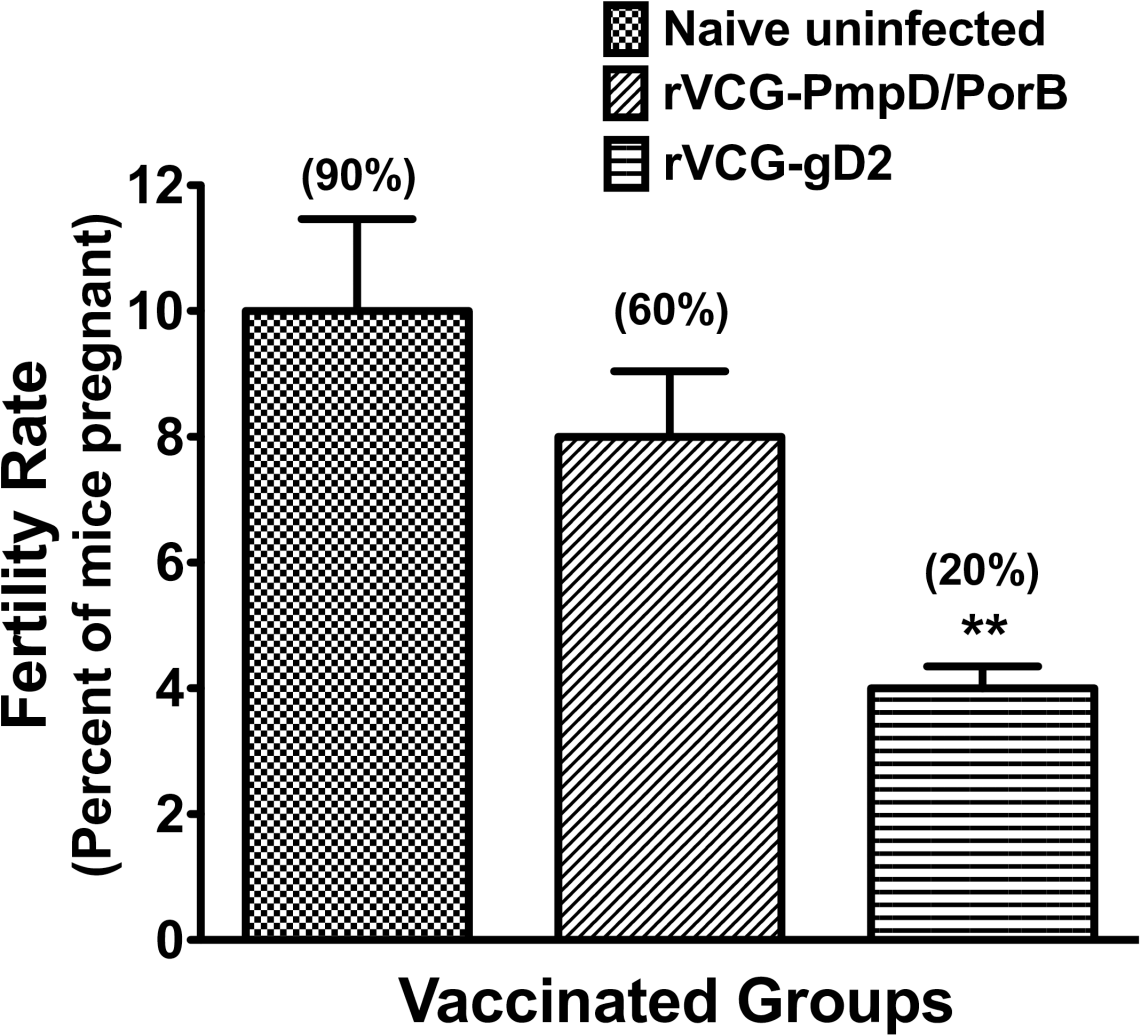
Protection against *Chlamydia*-induced infertility in immunized mice reinfected with live serovar E chlamydiae. Immunized mice were mated with proven fertile males four weeks after reinfection with live serovar E chlamydiae. The number of mice pregnant and the number of embryos recovered from each mouse/group was recorded, and the fertility rates were calculated. The data shows the percentage number of mice pregnant and the fertility rate, the mean number of embryos recovered from pregnant mice in each vaccinated group. Statistical significance was assessed by the Fisher exact test (in GraphPad Prism 5) in comparison with controls.

To assess *Chlamydia*-induced infertility in immunized and challenged mice, the number of mice pregnant and the mean number of embryos per group (fertility rate) were compared with those of uninfected age-matched control mice. The results revealed that the rVCG-PmpD/PorB-immunized mice retained significant fertility; six (60%) of the mice were pregnant with a fertility rate of 8 compared to nine (90%) of age-matched uninfected mice with a fertility rate of 10 (Fig 5). In contrast, the rVCG-gD2 control group showed significant loss of fertility, with two (20%) of mice pregnant and a fertility rate of 4. Representative reproductive tracts of pregnant mice from the rVCG-PmpD/PorB and uninfected control groups, shows multiple embryos (S1 Fig).

### IR immunization with rVCG-PmpD/PorB protects against *Chlamydia*-induced tubal pathology

**Fig 6:**
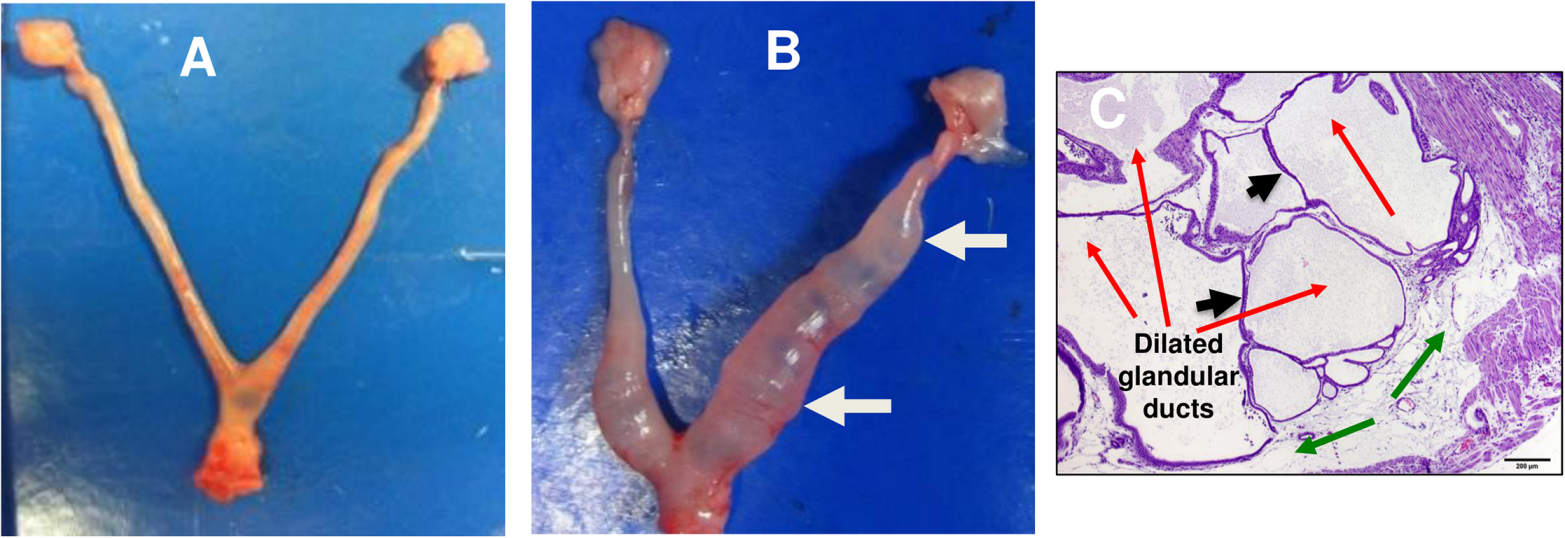
Protection against uterine inflammation in mice reinfected with live serovar E chlamydiae. Excised genital tracts of the non-pregnant mice from vaccinated and control groups were evaluated for the development of tubal pathologies and histological sections were stained with hematoxylin and eosin (H&E) and evaluated microscopically for tubal inflammation. Representative uterine horns of (A) Uninfected control or rVCG-PmpD/PorB vaccine-immunized mice with no visible pathology and (B) rVCG-gD2 control-immunized mice showing evidence of bilateral uterine horn lumenal dilation (white arrow). H&E-stained uterine tissue with multiple dilated glandular ducts of varying severity (C). Red arrows indicate the cross sections of dilated glandular ducts, which have pushed into the uterine horn lumen resulting in uterine horn lumenal blockades at multiple sites (arrowheads) and green arrows indicate dilated uterine lumen.

All non-pregnant mice from vaccinated and control groups were evaluated for the development of tubal pathologies. Microscopically, lesions were recorded as either absent or present. If absent (i.e., histologically normal), a score of 0 was assigned. If present, the severity of the lesions was recorded as minimal, mild, moderate, or severe, with pathology lesion severity scores of 1 through 4 for all animals (S1 Table). The group pathological lesion distribution was recorded as focal, multifocal, or diffuse, with distribution scores of 1, 2, or 3, respectively (S2 Table). The calculated total group severity and distribution scores were 40 for rVCG-PmpD/PorB and 151 for rVCG-gD2 while the mean group severity and distribution scores were 20 for rVCG-PmpD/PorB and 37.8 for rVCG-gD2 groups (S2 Fig). Severe uterine horn lumenal distension and glandular duct dilation were observed in the rVCG-gD2 infected group compared to none of the age-matched uninfected and rVCG-PmpD/PorB groups (Fig 6A–6C). However, none of the non-pregnant mice from both vaccinated and control groups of mice examined showed oviduct dilation or swelling (hydrosalpinx).

Microscopic evaluation of H&E-stained uterine tissue from the rVCG-gD2-immunized mice confirmed the uterine horn lumenal distension and glandular duct dilation observed by visual examination. Sections showed multiple dilated glandular ducts of varying severity that have pushed into the uterine horn lumen resulting in uterine horn lumenal blockades at multiple sites (Fig 6C).

### Histopathology of reproductive tissues

**Fig 7:**
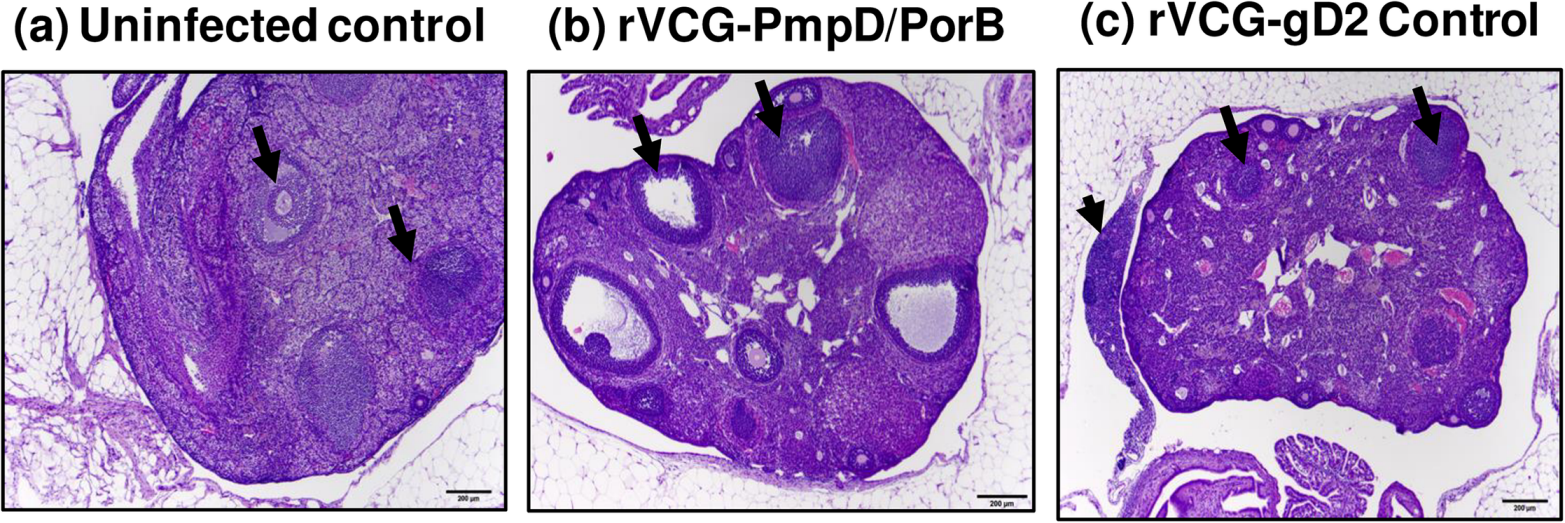
Histopathology of ovarian tissues from vaccinated and control mice reinfected with live serovar E chlamydiae. The H&E-stained sections of excised ovarian tissues were evaluated microscopically for distribution of inflammation and pathologic lesions. Representative images of ovarian tissues with normal multiple follicles (arrows) from (a) Uninfected control, (b) PmpD/PorB vaccine-immunized, and (c) rVCG-gD2-immunized mice (also showing periovarian inflammation). Arrowhead depicts a densely cellular collection of lymphocytes within the mesovarium. Scale bar, 200 microns.

**Fig 8:**
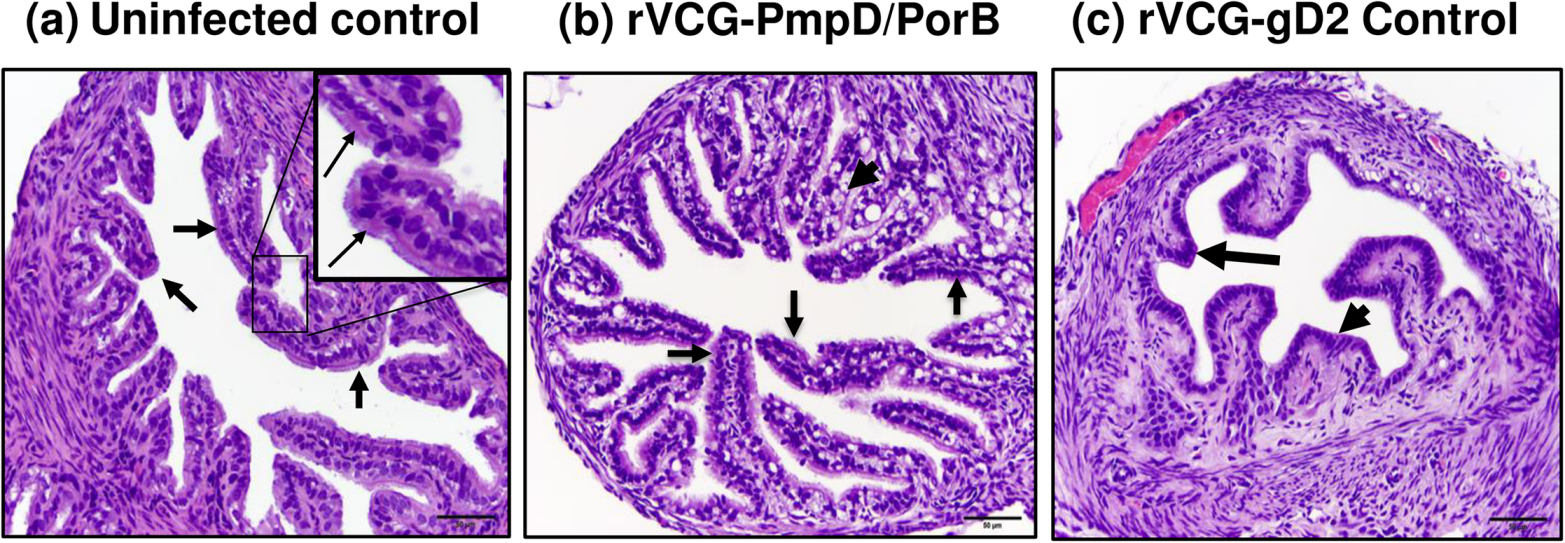
Histopathology of oviduct tissues from vaccinated and control mice reinfected with live serovar E chlamydiae. The H&E-stained sections of excised oviduct tissues were evaluated microscopically for distribution of inflammation and pathologic lesions. Representative images of oviducts from (a) Uninfected control mice with normal oviducts (arrows); insert shows enlarged section of villi with abundant cilia (thin arrows), (b) rVCG-PmpD/PorB vaccine-immunized mice showing intact normal villi with abundant cilia (arrows) but with numerous vacuoles (arrowhead), and (c) rVCG-gD2-immunized mice showing diffusely attenuated villi (arrow); there is villus atrophy and most of the cilia from the apical surfaces of the lining epithelia are absent (arrowhead). Scale bar, 50 microns.

**Fig 9:**
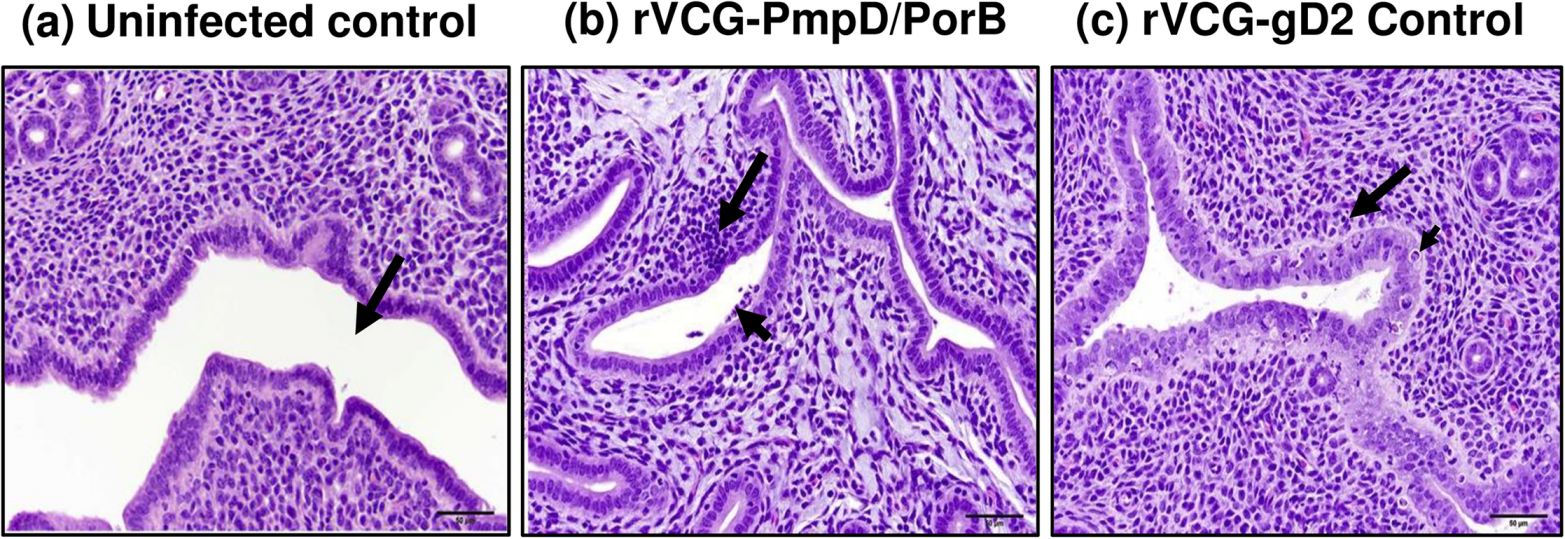
Histopathology of uterine tissues from vaccinated and control mice reinfected with live serovar E chlamydiae. The H&E-stained sections of excised uterine tissues were evaluated microscopically for distribution of inflammation and pathologic lesions. Representative images of uteri from (a) Uninfected control mice showing normal tissues, including an intact uterine lumen (arrow), (b) rVCG-PmpD/PorB vaccine-immunized mice showing mild inflammation characterized by a collection of lymphocytes which obscure the endometrium focally (arrow) and in the lumenal surface (arrowhead), and (c) rVCG-gD2 control-immunized mice showing diffusely hypercellular endometrium and lymphocytes in the lamina propria (arrow); arrowhead depicts an apoptotic cell within the thickened lamina epithelialis mucosa. Scale bar, 50 microns.

The H&E-stained sections of excised reproductive tract tissues were evaluated microscopically for distribution of inflammation and pathologic lesions. The H&E-stained sections of ovarian tissues of vaccinated and control mice reinfected with live serovar E chlamydiae are shown in Fig 7. The ovarian tissues of rVCG-PmpD/PorB-immunized mice appeared normal with multiple follicles comparable with those of age-matched uninfected control mice (Fig 7A and 7B). No inflammatory infiltrates were found in the oviduct tissues of these mice. In contrast, non-pregnant rVCG-gD2 control-immunized mice showed chronic inflammation in the periovarian region characterized by a densely cellular collection of lymphocytes within the mesovarium (Fig 7C). The micrographs in Fig 8A–8C) show representative examples of the oviduct tissues of infected and age-matched uninfected control mice. The oviduct tissues of rVCG-PmpD/PorB-immunized and age-matched uninfected control mice had normal villi with abundant cilia (Fig 8A and 8B). In contrast, there was moderate multifocal atrophy of villi, with segmental loss of apical cilia and vacuolar (fatty) degeneration in all the non-pregnant rVCG-gD2-immunized mice (Fig 8C). Except for the presence of minimal to mild acute uterine inflammation, the uteri in rVCG-PmpD/PorB-immunized mice were comparable to that of age-matched uninfected mice (Fig 9A and 9B). In contrast, the endometrium in rVCG-gD2-immunized mice was diffusely hypercellular, with foci of lymphocytic exocytosis and increased numbers of lymphocytes and apoptotic cells in the lamina propria (Fig 9C). Taken together, these results show that in addition to inducing protection against *Chlamydia*-induced infertility, rectal immunization could also significantly reduce the induction of tubal pathology in mice.

## Discussion

The route of vaccine administration plays an important role in the induction of immune effectors and their homing to the site of infection. *Chlamydia* immunity is dependent upon both T cell-mediated and humoral immune effectors. Therefore, a potentially effective route of vaccine administration will result in the induction of immune responses in both mucosal and systemic tissues. We tested the hypothesis that rectal mucosal immunization with a subunit vaccine delivered on the VCG platform would cross-protect mice against heterologous genital *Chlamydia* infection and upper genital tract pathologies. The rectal route of vaccine administration is attractive for vaccinating against genital chlamydial infection because it offers mucosal immunization that may exploit the common mucosal immune system to target immune effectors to mucosal pathogens like *C*. *trachomatis*. In addition, the rectal route obviates the concerns about the reactivity of some nasally administered adjuvants that may potentially cause neurological side effects in humans [14]. Thus, the rectal route has been proposed as an alternative mucosal route for immunization against diverse microbial pathogens [15–19]. Moreover, rectal immunization with a Chlamydial ghost-based vaccine was very effective in inducing immunity against enterohaemorrhagic *Escherichia coli* (EHEC) O157:H7 following heterologous challenge [20].

In the present study, immunologic evaluation revealed that rectal immunization of mice with rVCG-PmpD/PorB induced a cellular immune response characterized by high levels of chlamydial-specific CD4+ T cells that secreted IFN-gamma, TNF-alpha, IL-2 and IL-10 as well as IL-17. Also, the immune T cells proliferated significantly in response to *in vitro* restimulation with the heterologous *C*. *trachomatis* serovar E antigen. CD4+ T cells have been demonstrated in both human clinical and experimental animal model studies to play a key role in *C*. *trachomatis* immunity [31, 32]. IL-17 secreting Th17 cells have previously been shown to play a role in protection against *C*. *muridarum* respiratory infections [33] and other intracellular pathogens [34]. A recent study showed that although IL-17 promotes vaginal chlamydial clearance, it also promotes immunopathological tissue damage [35, 36]. However, other studies strongly suggest that IL-17 impairs chlamydial clearance and plays a role in the induction of upper genital tract pathology [35, 36]. In another study [37], IL17a knockout mice were shown to have significantly less chlamydial burden and less pathology compared to wild type mice. Our results also show that IR immunization elicited significant cross-reactive local mucosal and systemic IgA and IgG2c antibody responses that were detectable in serum and vaginal secretions. Although the precise role of antibodies in the resolution of primary *Chlamydia* infection remains incompletely understood [38, 39], the induction of the Th1-associated IgG2a (the BALB/c homolog of IgG2c) isotype has previously been shown to be associated with protection against *Chlamydia* [40]. The higher magnitude of specific Th1-associated IgG2c antibodies in genital secretions compared to IgA is consistent with findings in humans showing that the dominant immunoglobulin isotype found in the cervico-vaginal fluid of the female genital tract is IgG rather than secretory IgA [41, 42]. Thus, the humoral immune effectors that mediate chlamydial clearance in the genital tract likely originate from immune inductive sites in the systemic circulation rather than the genital tract.

Vaccine efficacy analysis showed that IR immunization with rVCG-PmpD/PorB vaccine resulted in a significant reduction in the number of mice shedding chlamydiae that was comparable to mice previously infected with live chlamydiae. In addition, rVCG-PmpD/PorB vaccine recipient mice exhibited a lower intensity and duration of chlamydial shedding compared to rVCG-gD2 controls. We previously showed intramuscular (systemic) immunization with a VCG-based chlamydial vaccine induced protective immunity in mice against genital challenge with *C*. *muridarum* and *C*. *trachomatis* serovar D [11, 12]. The significant reduction in the intensity and duration of infection in rVCG-PmpD/PorB vaccine-immunized mice observed in the current study highlights the potential of rectal mucosal immunization in the induction of protective immunity in the female genital tract.

In addition to inducing broad-based protective immunity against multiple serovars, an efficacious chlamydial vaccine should also protect against development of upper genital tract pathology, particularly infertility. Infertility is the most significant outcome of *Chlamydia* genital infection in humans and is one of the quantitative measures used to assess protection against upper genital tract disease. A previous study demonstrated that a single intravaginal inoculation with 10^7^ IFU of *C*. *trachomatis* serovar E induced upper genital tract pathology in C57BL/6 mice [24] although the authors did not investigate infertility. In another study, Ramsey, KH and colleagues reported that repeat infection of mice with 1 x 10^7^ IFUs of *C*. *trachomatis* serovar E resulted in an increased pathological outcome, with 60% of the mice becoming infertile [26]. Since recurrent chlamydial infections have been reported to increase the risks for upper genital tract pathology, such as ectopic pregnancy and pelvic inflammatory disease [43–45], we used repeat intravaginal inoculation with a high dose of *C*. *trachomatis* serovar E to stimulate upper genital tract pathology. Our study showed *Chlamydia*-specific immune effectors elicited by either a previous infection or vaccination induced a significant degree of protective immunity marked by a lower intensity and shortened duration of infection. It is noteworthy, however that vaccinated mice were protected from infertility (fertility rate, 8) whereas control-immunized mice were not (fertility rate, 2). These results confirm our previous findings indicating that vaccine- but not infection-induced immunity protects against *Chlamydia*-induced upper tract pathology [46, 47]. The implication of all these results is that although a live *Chlamydia* infection results in significant clearance of chlamydiae in the lower genital tract, this does not lead to protection against upper genital tract disease.

Another significant finding in the current study is the occurrence of uterine horn lumenal dilation or distension and mucosal edema that were commonly observed in the rVCG-gD2 control mice, which were immunized with a chlamydial irrelevant antigen and reinfected with *C*. *trachomatis* serovar E. Our results confirm the findings of a previous study that reported destruction of the uterine horn epithelium with marked mucosal edema and distinct distention of the uterine horns following intravaginal infection of BL6 mice with *C*. *trachomatis* serovar E [24]. The uterine horn lumenal dilation correlated with the glandular duct dilation detected microscopically. The uterine horn dilation was chronic and therefore likely caused by glandular duct dilation, which may occur first, pushing into the uterine horn lumen causing uterine horn lumenal blockades at multiple sites and leading to extensive uterine horn lumenal dilation as previously reported following *C*. *muridarum* infection [48]. Although hydrosalpinx is an important pathologic marker of *Chlamydia***-**induced infertility in mice [6, 48–50], none of the immunized and control mice in the studies reported here developed any overt hydrosalpinx as assessed by visual examination. It must be noted however, that directly comparing the incidence of *Chlamydia*-induced upper genital tract pathology in mouse models to that of natural infection of women is potentially problematic. This is because a number of confounding variables, including host genetic factors, hormonal secretions and antibiotic treatment at the early stages of infection greatly influence the progression of chlamydial disease in both humans and animal models. Although *C*. *trachomatis* genital tract infection of mice, like *C*. *muridarum*, is not a perfect model of human chlamydial genital infection, it is a reasonable model for the study of *Chlamydia*-host interactions and together with the *C*. *muridarum* model has contributed to our expanding knowledge of chlamydial disease in humans.

Interestingly, while rVCG-PmpD/PorB vaccine-derived immune effectors prevented oviduct and uterine inflammation, rVCG-gD2 control-immunized mice were not protected. This clearly indicates that chlamydial burdens in the lower genital tract do not correlate with upper genital tract disease status in mice infected with live *Chlamydia*. In general, tissue alterations in in rVCG-PmpD/PorB vaccine-immunized mice where present, were minimal and of lower incidence and severity with an average severity-and-distribution score of 20, which is almost 50% lower than that of rVCG-gD2 control-immunized mice (37.5). Oviduct and uterine horn pathology involving acute inflammatory response, induced by primary infection with *C*. *trachomatis* serovar E have previously been reported [24]. That study suggested a role for the acute inflammatory response in the development of tissue damage. More recently, intravaginal infection of mice with *C*. *trachomatis* serovar D was shown to induce endometrial leukocyte infiltration that was significantly (p < 0.05) more intense on day 7 compared to day 90 postinfection [35]. Moreover, repetitive genital exposure of mice to *C*. *trachomatis* serovar D induced cystic changes and profound uterine lumen distension not detected in uninfected, age-matched controls [35] as observed in our studies with serovar E. Inflammation is thought to be the major cause of chronic fallopian tube tissue damage seen in infertile women infected with *C*. *trachomatis* [51]. Studies in animal models also suggest CD8 T cells and other immune mechanisms play a major role in *Chlamydia*-induced upper tract pathology, including infertility [47, 52].

## Conclusions

This study demonstrates that IR immunization elicited immune effectors that mediate chlamydial clearance in the genital tract, indicating cooperation between the rectal and genital mucosae functioning as mucosal inductive and effector sites, respectively, according to the compartmentalization within the common mucosal immune system. Our study also confirmed that infection- and vaccine-induced immunity are functionally distinct in their ability to prevent the sequelae of chlamydial infection such as infertility and upper genital tract inflammation. Taken together, these results highlight the potential of rectal delivery as a viable mucosal route for eliciting protective immunity in the female genital tract.

## Supporting information

S1 Fig Reproductive tracts of pregnant mice. Representative reproductive tracts of pregnant mice from the rVCG-PmpD/PorB and the aged-matched uninfected control groups showing multiple embryos.
(TIF)

S2 Fig Protection against uterine inflammation in rVCG-gD2-immunized mice reinfected with live serovar E chlamydiae. rVCG-gD2-immunized mice reinfected with serovar E chlamydiae were not protected from genital tract inflammation, such as uterine lumenal dilation (A) and mucosal edema (B). Mean lesion severity and distribution scores (C) were significantly higher (*p* ≥ 0.05) in mice immunized with rVCG-gD2 than those immunized with rVCG-PmpD/PorB.
(TIF)

S1 Table Number of animals with tubal pathology lesions. Lesion diagnosis in the ovaries, oviducts and uteri of mice immunized with rVCG-PmpD/PorB or rVCG-gD2 control after reinfection with serovar E chlamydiae.
(TIFF)

S2 Table Pathological lesion severity and distribution scores. Severity and distribution of pathological lesions in the ovaries, oviducts and uteri of mice immunized with rVCG-PmpD/PorB or rVCG-gD2 control after reinfection with serovar E chlamydiae.
(TIF)
