# Supplementary figures and images for: Rectal administration of a chlamydial subunit vaccine protects against genital infection and upper reproductive tract pathology in mice
Source: PLoS One. 2017 Jun 1;12(6):e0178537. doi: 10.1371/journal.pone.0178537 (PMC5453548; doi:10.1371/journal.pone.0178537)

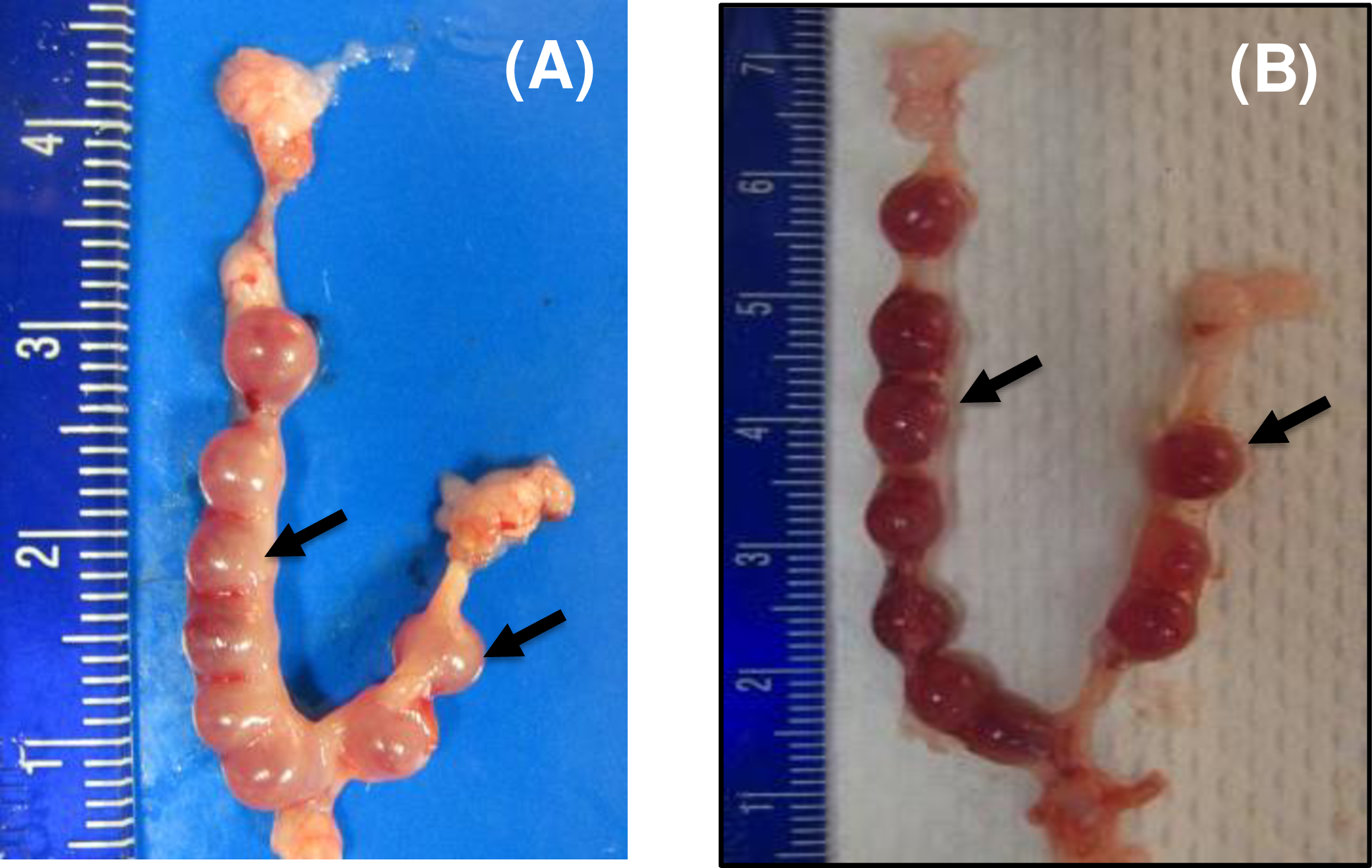

Supplement: S1 Fig — Representative reproductive tracts of pregnant mice from the rVCG-PmpD/PorB and the aged-matched uninfected control groups showing multiple embryos. (TIF) [file pone.0178537.s001.tif]

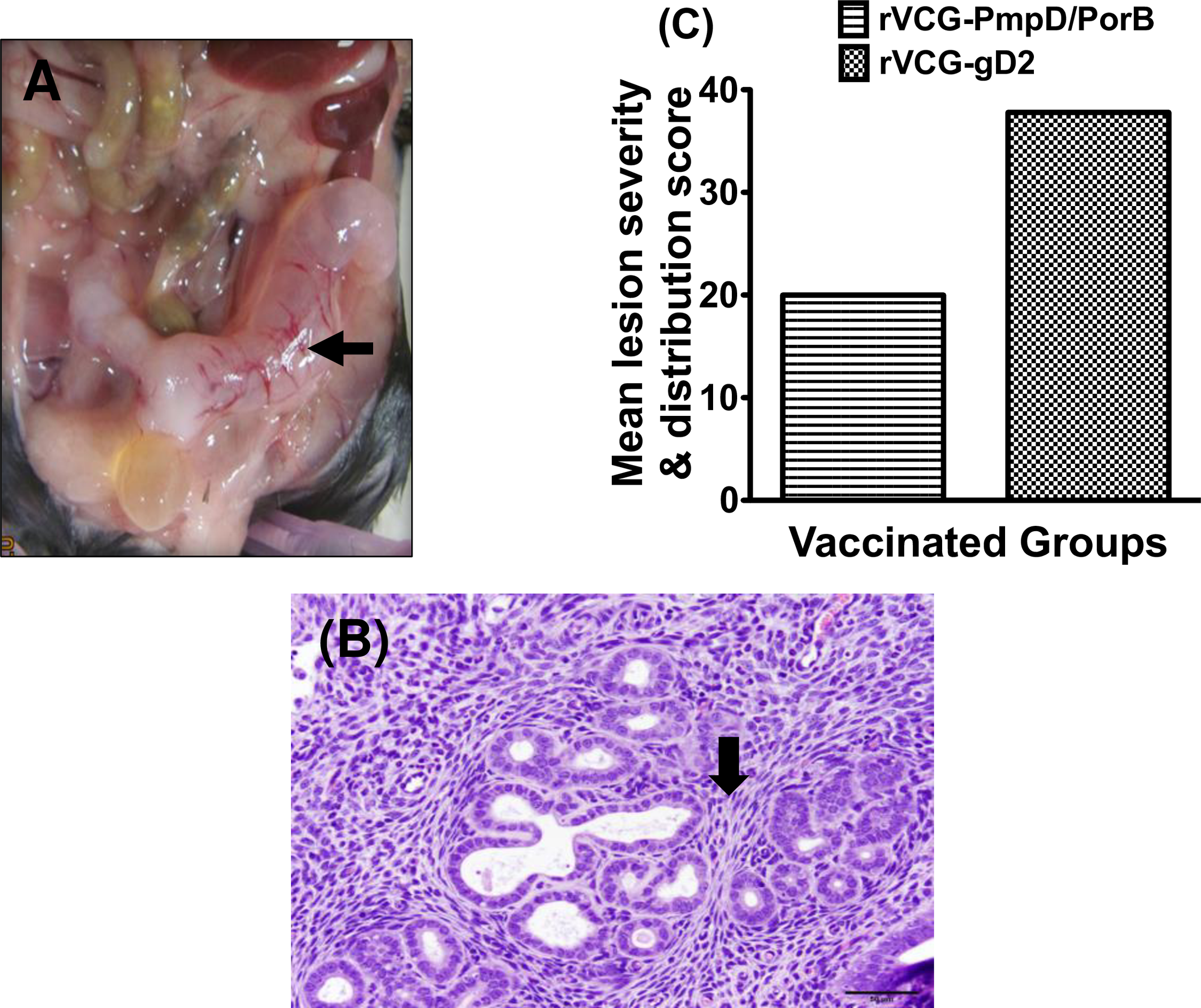

Supplement: S2 Fig — rVCG-gD2-immunized mice reinfected with serovar E chlamydiae were not protected from genital tract inflammation, such as uterine lumenal dilation (A) and mucosal edema (B). Mean lesion severity and distribution scores (C) were significantly higher (p ≥ 0.05) in mice immunized with rVCG-gD2 than those immunized with rVCG-PmpD/PorB. (TIF) [file pone.0178537.s002.tif]

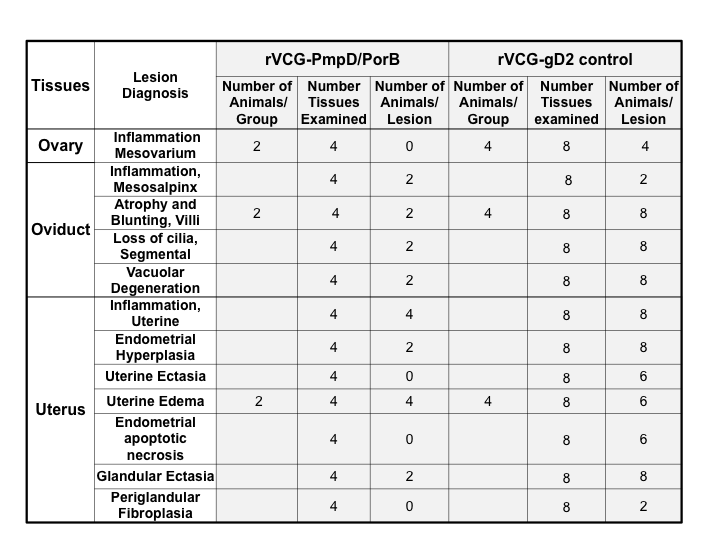

Supplement: S1 Table — Lesion diagnosis in the ovaries, oviducts and uteri of mice immunized with rVCG-PmpD/PorB or rVCG-gD2 control after reinfection with serovar E chlamydiae. (TIFF) [file pone.0178537.s003.tiff]

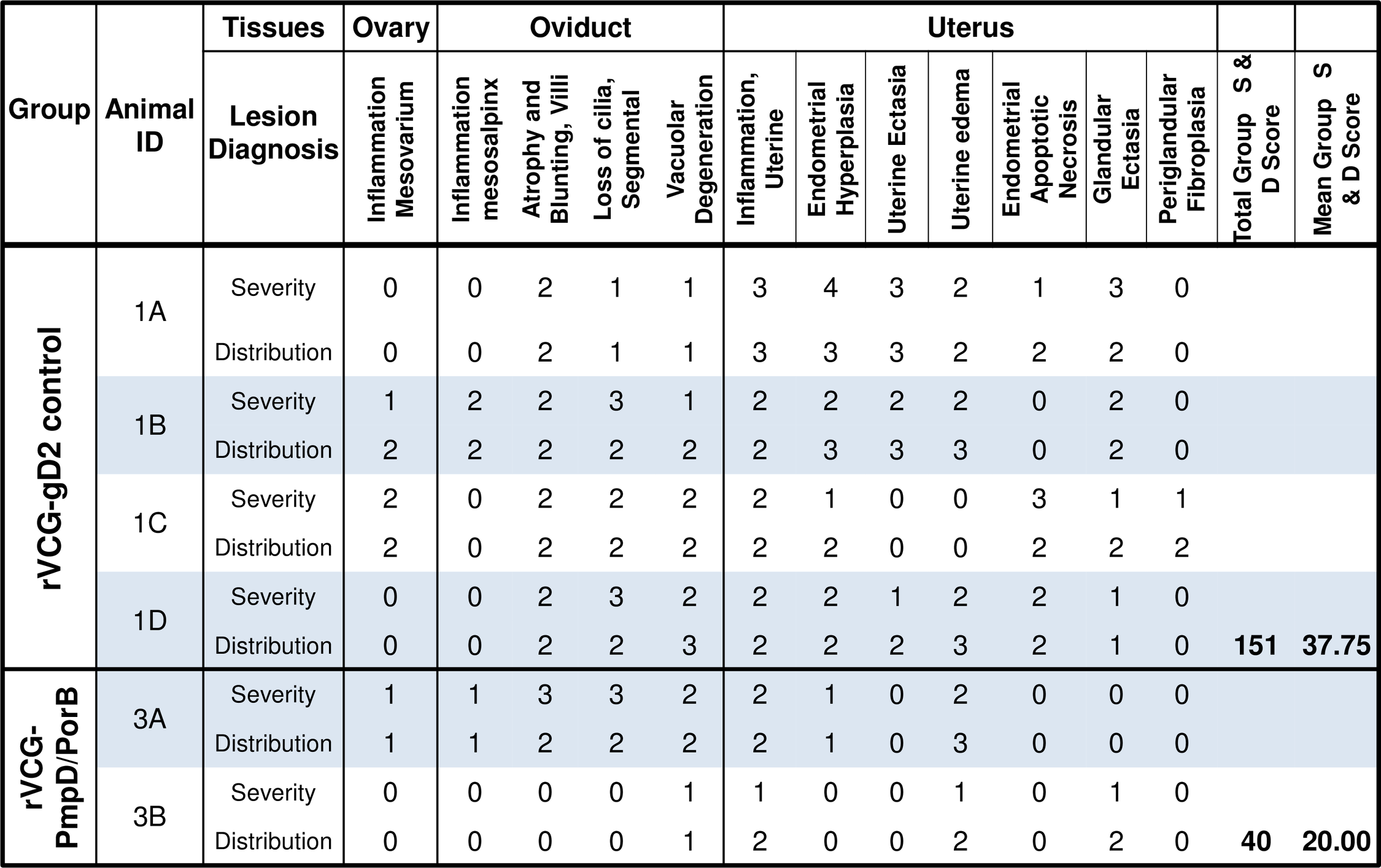

Supplement: S2 Table — Severity and distribution of pathological lesions in the ovaries, oviducts and uteri of mice immunized with rVCG-PmpD/PorB or rVCG-gD2 control after reinfection with serovar E chlamydiae. (TIF) [file pone.0178537.s004.tif]
